# Study on Corrosion Behavior of Porous Pure Copper Based on Electrochemistry and Scanning Kelvin Probe

**DOI:** 10.3390/ma16237370

**Published:** 2023-11-27

**Authors:** Xuedan Chen, Qilong Liao, Hanyang Zuo, Qingshan Fu

**Affiliations:** 1 School of Materials and Chemistry, Southwest University of Science and Technology, Mianyang 621010, China; chenxdzg@126.com; 2 College of Material Science and Engineering, Sichuan University of Science and Engineering, Zigong 643000, China; zuohanyang2017@163.com; 3 Vanadium and Titanium Resource Comprehensive Utilization Key Laboratory of Sichuan Province, Panzhihua 617016, China

**Keywords:** porous copper, scanning Kelvin probe, electrochemical impedance, dynamic electrolyte

## Abstract

Porous metals are widely used in filtration and separation, flame retardant explosion-proof, biomedical application, etc. Compared with its corresponding dense metal, the presence of porous structures also leads to different corrosive performances in porous metal. Some studies have utilized the weight loss method, electrochemical impedance to evaluate porous metal corrosion behavior; however, the influence of pore structure on metal corrosion is still ambiguous, and present methods used for analyses of porous metal corrosion are statistical averages of the corrosion behavior of the entire porous material, which cannot accurately reflect the corrosion behavior inside the pores. Herein, we prepare the porous copper samples with 0, 24, 72, and 96 pores using a mechanical process, and employ scanning Kelvin probe combined with electrochemical polarization and impedance spectroscopy to test the corrosion performance of the porous copper in static and dynamic NaCl solutions. The relevant results indicate that in the static solution, the corrosion resistance of the samples gradually increases with the rise in the number of pores. By contrast, in the dynamic solution, the 24-pore sample is more susceptible to corrosion than the sample without the pore.

## 1. Introduction

Porous metal materials, due to their special properties [1,2,3,4], have been widely used in various fields, such as filtration and separation [5], fluid penetration and distribution control [6], efficient combustion [7], mass and heat transfer [8], damping and acoustic adsorption [9,10], and flame retardant [11]. The characteristics of pore structure, including pore size, porosity, pore shape, open or closed cell, and uniformity of pore distribution, determine the performances of porous metals [4,12,13,14]. Generally, a certain pore structure is required to meet a special application in different fields [15,16,17].

Pore structure endows unique physical and chemical properties to porous metals, simultaneously brings some adverse effects [18]. Among them, deterioration of corrosion resistance is caused by the open and discontinuous internal structure in porous metals [19,20,21]. To understand corrosion in porous metals, different methods are employed. Wu et al. [22] established a corrosion kinetic curve by the weight loss method to study the corrosion resistance of porous titanium and aluminum metal materials in hot hydrochloric acid at pH = 2 and pH = 3. The weight loss method [23] was also used to study the effect of the pore structure on the corrosion resistance of closed-cell aluminum foam and the relevant results point out that the corrosion resistance of porous aluminum foam is much lower than that of dense aluminum material. With similar pore sizes, increasing porosity causes a rise in surface area and a decrease in corrosion resistance. The corrosive behavior of plasma-sprayed porous titanium in dynamic and static electrolyte was determined by potentiodynamic polarization and electrochemical impedance, and suggests that the porous titanium has weaker corrosion resistance compared with solid titanium material [24]. Some 3D printed porous metals were also subjected to corrosion evaluation, and the results also indicate that the introduction of pores leads to aggravation of corrosion [25].

According to the aforementioned research, the methods for evaluating the corrosion behavior of porous metal materials mainly depend on the weight loss method, electrochemical impedance and potentiodynamic polarization. Specially, the use of the absolute impedance at a low frequency to determine the corrosion resistance is widely used [26]. These methods are based on the statistical averages of the corrosion behavior of the entire porous material, which cannot accurately reflect the corrosion behavior inside the pore structure and the corrosion difference between the outer and inner surfaces of the pores. Therefore, this work utilizes scanning Kelvin probe (SKP) to study the corrosion behavior of porous copper materials in NaCl solutions. The inner surface of the pore is scanned by a micro-Kelvin probe, and the microscopic electrochemical information is collected, to which the corrosion law inside the pores is analyzed according. We believe the relevant results can provide a reference for the design of the corrosion-resistant structure of porous copper in the corrosive medium.

## 2. Materials and Methods

### 2.1. Materials

The pure copper T2 sheet used in the experiment was purchased from commercial routes, and its composition is shown in Table 1. Sodium chloride (NaCl, AR) and absolute ethyl alcohol were purchased from Cologne Chemicals Co., Ltd., Chengdu, China. Laboratory-made deionized water was used throughout the experiment.

### 2.2. Preparation of Porous Pure Copper Electrodes

**Figure 1:**
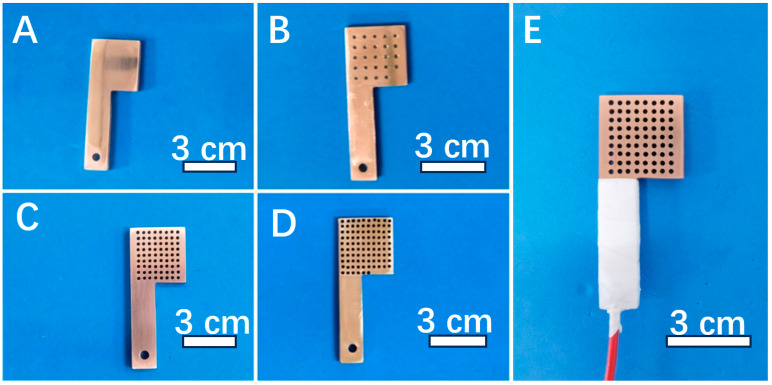
Photos of pure copper samples: (**A**) no pore, (**B**) 24 pores, (**C**) 72 pores, (**D**) 96 pores and (**E**) sample sealed with silicone.

The pure copper sheet was processed into L-shaped specimens (rectangular part + square part), as shown in Figure 1 by a diamond wire cutting machine (STX-605, Kejing Auto-Instrument CO., LTD,.Shengyang, China), and different numbers (24, 72, 96) of pores with a diameter of 1 mm were produced in their square part by mechanical working. Then, a hole was drilled in the rectangular part for connecting with copper wire. Then, the surface of the specimen and the inside of the pores were polished with 200#, 400#, 600#, 800#, 1200#, 1500#, 2000# abrasive paper and silicon-carbide-spray in turn to realize mirror surface. The polished specimens were ultrasonically cleaned in deionized water and anhydrous ethanol and dried with cold air. The blow-dried specimens were connected to copper wire and sealed with silicone, exposing the square part as the working surface (20 mm × 20 mm × 3 mm), as shown in Figure 1E.

### 2.3. Electrochemical Polarization Curve and the Impedance Spectroscopy Test

The three-electrode system was adopted in the electrochemical test. A porous copper sample was used as the working electrode. A saturated calomel electrode, a platinum electrode and 3.5 wt% of NaCl solution were employed as the reference electrode, the auxiliary electrode and the electrolyte, respectively. The electrochemical tests were carried out on an electrochemical workstation (CHI660E, Chenhua Instruments Co., Shanghai, China) at 25 °C. Firstly, the open circuit potential was tested until to achieve a stable value, and then the electrochemical polarization curve was measured in the potential scanning range of −250–650 mV at a scanning speed of 1 mV/s; the electrochemical impedance spectroscopy (EIS) was tested in the frequency range of 0.01~100 kHz with the self-corrosion potential as the starting potential. The AC signal amplitude is 5 mV. The resultant impedance spectra were analyzed by ZsinpWin software v3.60. To ensure the reliability of the data obtained, at least three parallel samples were measured per set of experiments. In order to evaluate the influence of electrolyte flow on the corrosion behavior of the porous copper, a dynamic circulation system designed in the previous research work [24] was used to make the electrolyte flow at a speed of 0.17 m/s.

### 2.4. Scanning Kelvin Probe Tests

**Figure 2:**
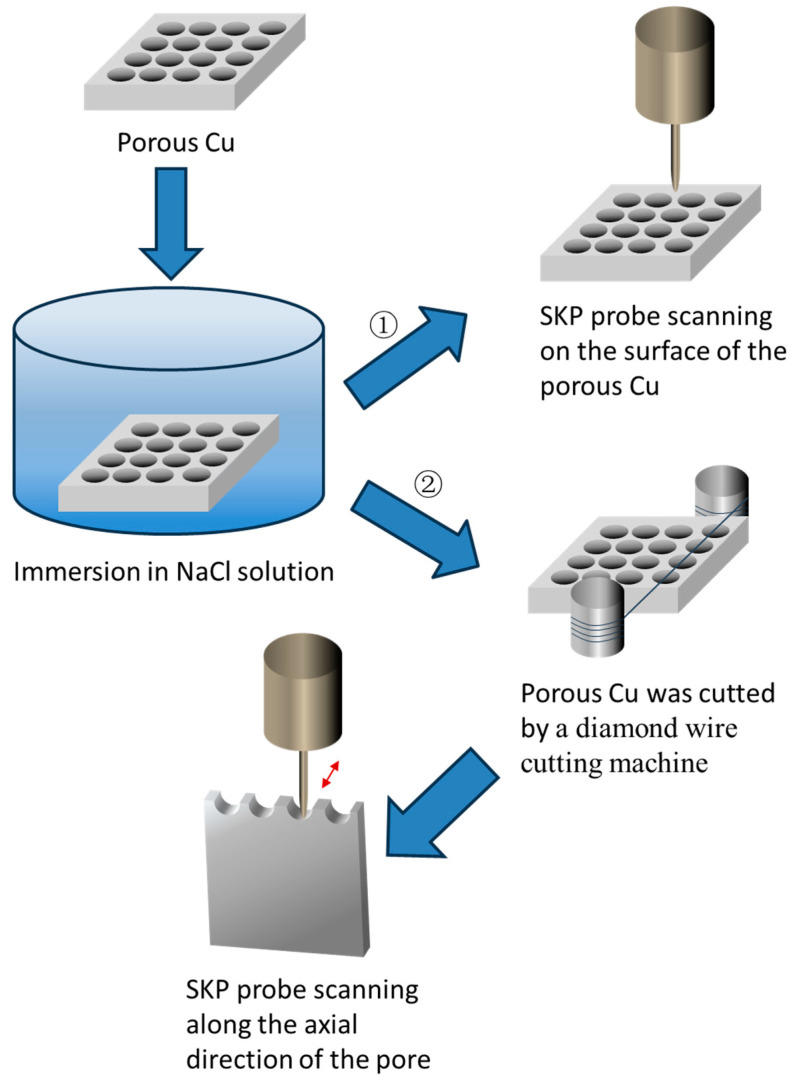
Schematic illustration of the process for corrosion evaluation by SKP. (➀ stands for the route of scanning on the surface of the samples; ➁ stands for the route of scanning on the inner surface of the pores).

A scanning Kelvin probe assembled on the Micro-Area Scanning Electrochemical Workstation (VersaSCAN, Ametec, Berwyn, PA, USA) was used to study the corrosion behavior of the outer surface and inner of the pores. The Kelvin probe is a non-contact, non-destructive test method that measures the work function difference between the material and the probe to obtain the surface potential distribution of the specimen [11]. Before testing, the porous copper electrode was fixed on the sample stage of the workstation (as shown in Figure 2) which was placed in an electrolytic cell.

For the test of potential distribution on the outer surface, the sample was immersed in NaCl solution for a certain period and then was taken out and blow-dried using cold air. According to the route one in Figure 2, the surface of the dried sample was scanned by the SKP probe in an area-scanning mode keeping the tip (of the probe)-surface (of the sample) distance of 100 μm in a scanning step of 50 μm. The scanning area (with the size of 3000 μm × 3000 μm) was positioned between two pores. The specimen without immersion in NaCl solution was also tested.

**Figure 3:**
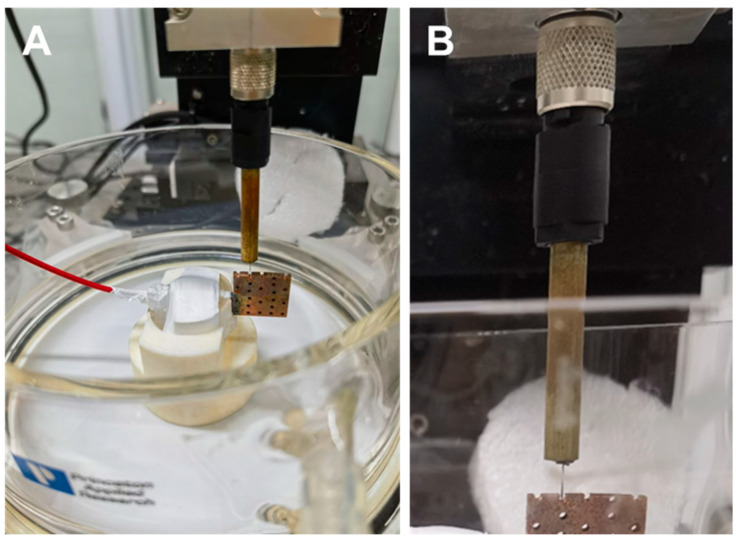
(**A**) Photo of truncated sample fixed on the sample stage of the micro-scanning electrochemical workstation with cross section facing the probe and (**B**) partial enlarged photo of (**A**).

When using SKP to test the potential distribution in the pores, porous samples were soaked in NaCl solution for a certain time, then the samples were taken out and were truncated by a diamond wire cutting machine to expose the inner surface of the pores according to the route two in Figure 2. The truncated sample was fixed on the sample stage with cross section facing the SKP probe, as shown in Figure 3. The probe tip was above 100 μm from the lowest point of the pore’s inner surface and moved along the axial direction of the pore. The scanning range was 200 μm × 3000 μm with a scanning step of 50 μm. The sample without immersion in NaCl solution was also tested.

### 2.5. Observation of Corrosion Morphology

Scanning electron microscopy (SEM, EVO MA15, Zeiss, Oberkochen, Germany) was used to characterize the surface topography of the porous pure copper specimens before and after immersion in 3.5 wt% NaCl solution. Before SEM test, the copper sample was washed with deionized water to remove the surface corrosion solution, and then was dried by cold air.

## 3. Results and Discussion

### 3.1. Potentiodynamic Polarization Curve

**Figure 4:**
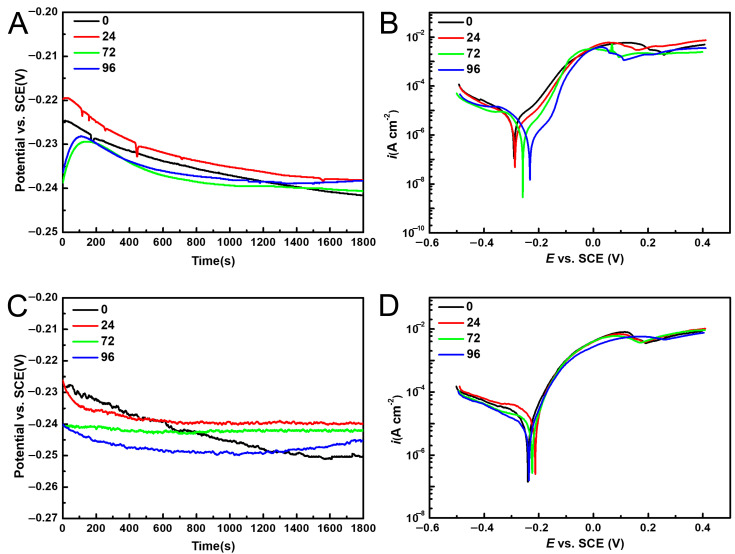
OCP plots of the specimens in the (**A**) static and (**C**) dynamic 3.5% NaCl solutions; polarization curves of the specimens in the (**B**) static and (**D**) dynamic 3.5% NaCl solutions.

The OCP curves are shown in Figure 4A,C, and the stable potentials are listed in Table 2. The polarization curves of pure copper specimens with different numbers of pores in the static and dynamic 3.5 wt% NaCl are shown in Figure 4B,D. Accordingly, the polarization curves show a similar shape, indicating that the corrosion mechanism of the pure copper samples did not change with the number of pores. The cathodic polarization reaction of the pure copper specimens was the reduction of oxygen; the dissolution reaction of the metal took place during the anode process, which can be divided into three stages: the active dissolution zone, the passivation zone, and the limit current region.

The polarization curves in Figure 4 were fitted using the Tafel curve extrapolation method, and the electrochemical parameters obtained are shown in Table 2. Accordingly, in static 3.5 wt% NaCl solution the corrosion potential (E*_corr_*) of the samples undergoes a slow positive shift as the number of pores increases. Generally, E*_corr_* reflects the corrosion tendency of metal materials, so the changes of E*_corr_* indicate that the corrosive possibility of the porous copper reduced as the rise in the pore’s number. Additionally, the value of corrosion current density (I*_corr_*) gradually decreases with the increase in the number of pores, and in 96-pore sample the value of I*_corr_* achieves the minimum indicating the best corrosion resistance. In the static solution, the corrosion resistance of the porous samples gradually was reinforced with the increase in the number of pores. In the dynamic solution, according to Table 2, the value of I*_corr_* first increases and then decreases. The maximum value of I*_corr_* (16.281 μA/cm^2^) is acquired in 24-pore sample, indicating this sample had the worst corrosion resistance in dynamic solution, which is different from the sample in the static NaCl solution.

### 3.2. AC Impedance Test

**Figure 5:**
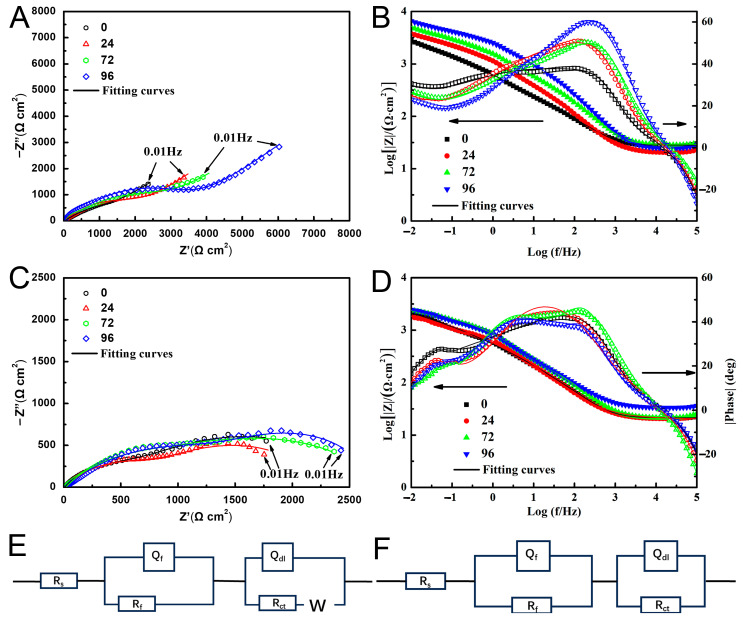
Nyquist plot (**A**) and Bode plot (**B**) of the samples in the static 3.5 wt% NaCl solution; Nyquist plot (**C**) and Bode plot (**D**) in the dynamic 3.5 wt% NaCl solution; (**E**,**F**) the equivalent circuit diagrams of (**A**,**C**), respectively.

The Nyquist plots and the Bode plots measured in the static conditions are show in Figure 5A,B. With the increase in the number of pores, the radius of the impedance curve in the high-frequency range increases, indicating that the corrosion resistance of the dense sample is better than that of the porous ones. All the impedance curves exhibit diffusion tails corresponding to the Warburg impedance in the low-frequency region. This indicates the occurrence of diffusion processes on the sample surface, including the diffusion of soluble metal ions from the sample facing the anode in solution and the dissolved oxygen diffusion towards the cathode in the opposite direction.

The Nyquist plots (Figure 5C) and the Bode plots (Figure 5D) in the dynamic solution are different from those in the static solution. The impedance spectra present two semicircular arcs and the Warburg arc in the low-frequency region disappeared, as shown in Figure 5C. With the increase in the number of pores, the radius of the capacitance arc first decreases and then increases. The radius of the capacitance arc of the 24-pore sample is the smallest, indicating the most serious corrosion in this sample.

According to the characteristics of the Nyquist diagram in Figure 5A,C, the equivalent circuit diagrams in Figure 5E,F were chosen to fit them, respectively. In the equivalent circuit diagrams the solution resistance (R_s_), the capacitance (Q_f_) and resistance (R_f)_ of the corrosion product layer, the electric double-layer capacitance (Q_dl_) and charge transfer resistance (R_ct_), the Warburg impedance (Z_w_) in the low-frequency region were used.

**Figure 6:**
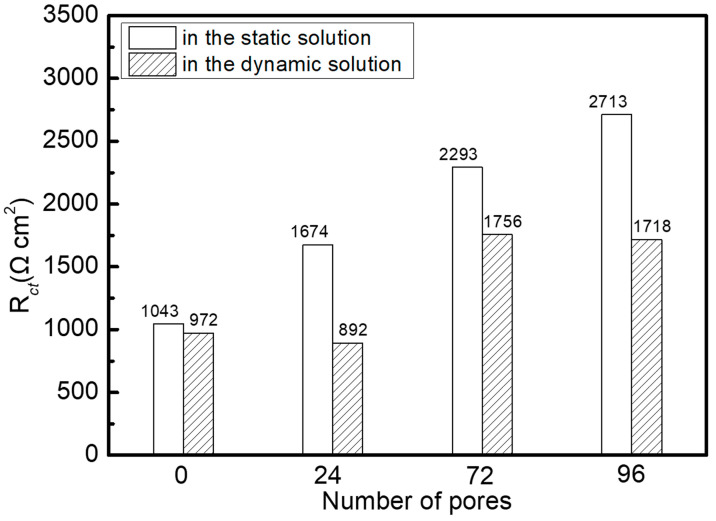
Charge transfer resistance of the samples in the static and dynamic solutions.

The electrochemical impedance related parameters obtained by fitting with ZSimpWin software v3.60 are shown in Table 3 and Table 4. The quality of the fitting is usually evaluated by the value of χ^2^. All the values of χ^2^ are less than 0.003, indicating the fitting data match the experimental results well. In Figure 6, in the static solution, the value of R_ct_ gradually increases with the rise in the number of pores; however, in the dynamic solution, the value of R_ct_ reaches a minimum in 24-pore sample. R_ct_ is the resistance of the charge transfer through the phase boundary between the metal and the electrolyte. The value of R_ct_ can reflect the rate of dissolution reaction of the metal. Accordingly, in the static solution the introduction of pores can boost corrosion resistance of all the porous sample and the maximum resistance capacity was harvested in the 96-pore sample. Nevertheless, in the dynamic solution, the 24-pore sample presented the worse corrosion resistance than sample without the pore. Furthermore, the samples with the same pores exhibit the higher values of R_ct_ in the static solution than in the dynamic solution. This means that perturbation of the solution leaded to the changes of corrosion behavior of the porous samples.

### 3.3. Analyses of the Scanning Kelvin Probe Test

SKP was used to measure the potential changes on the outer and inner surface of pores before and after immersion in the static 3.5 wt% NaCl solution for 14 days. Based on the principle of the SKP test, there is a linear relationship between the corrosion potential (E_corr_) and the Kelvin potential (E_kp_): E_corr_ = α + b E_kp_. Therefore, the highest Kelvin potential (E_kpmax_) and the lowest Kelvin potential (E_kpmin_) of the measurement system correspond to the cathode potential and anode potential of the reaction system, respectively. ${\bar{∆E}}_{kp}$ is defined as the average value of the difference between the maximum and minimum Kelvin potentials on the adjacent surfaces, which is the corrosion potential difference of the system, and can be used to evaluate the trend of corrosion occurrence.

**Figure 7:**
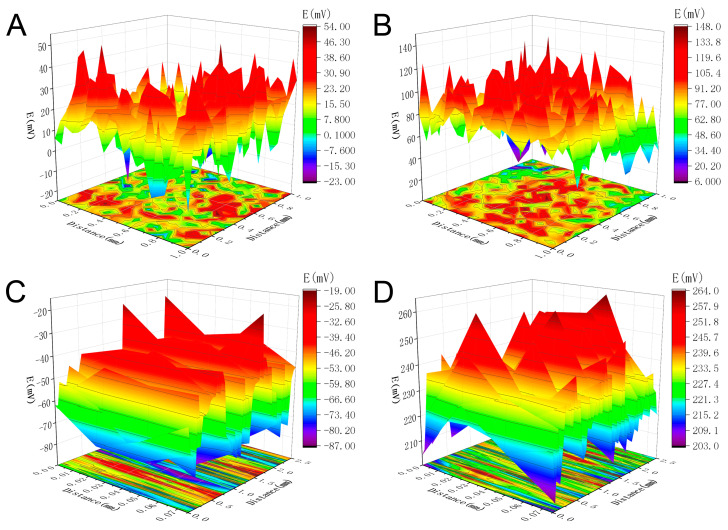
SKP potential distribution of the 24-pore porous copper on (**A**) outer surface and (**C**) inner surface of pore before immersion in the static 3.5 wt% NaCl for 14 d; (**B**) outer surface and (**D**) inner surface of pore after immersion in the static 3.5 wt% NaCl for 14 d.

As shown in Figure 7A, the E_kp_ distributes on the surface of the 24-pore sample uniformly with an average value of 25 mV before immersion of the sample in 3.5 wt% NaCl solution. The values of E_kpmax_, E_kpmin_ and ${\bar{∆E}}_{kp}$ are about 54 mV, −23 mV, and 57 mV, respectively. After immersion for 14 d, the potential of the outer surface changes significantly (Figure 7B). The average E_kp_ increases to 98 mV, and ${\bar{∆E}}_{kp}$ of 43 mV is observed. The decrease in potential difference indicates that the corrosion rate of the specimen drops, and the corrosive possibility decreases. With respect to the inner surface of the pores, the uniform distribution of E_kp_ is found before corrosion (Figure 7C). The average value of E_kp_ is −65 mV. ${\bar{∆E}}_{kp}$ 61 mV. After immersion in the NaCl solution, the value of E_kp_ presents obvious positive shift. The average E_kp_ is about 223 mV and ${\bar{∆E}}_{kp}$ is approximately 27 mV. The decrease in ΔE_kp_ indicates that after immersion in the electrolyte the corrosive possibility of the inner surface of pore declined due to formation and thickening of corrosion product layer. After immersion for 14 d, the value of ${\bar{∆E}}_{kp}$ of the outer surface is bigger than that of the inner surface of the pore, indicating the lower possibility to corrode on the inner surface of the pore. The reason is that the corrosion product is easy to accumulate and thicken in pores, which can relieve the attack of aggressive Cl^−^ to the metal matrix.

### 3.4. Morphologies of Samples before and after Immersion

**Figure 8:**
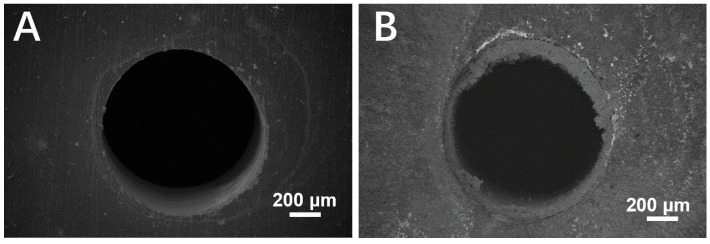
Morphology of the 24-pore porous copper (**A**) before and (**B**) after immersion in 3.5 wt% NaCl solution for 14 d.

It was observed by scanning electron microscopy that the surface morphologies of the 24-pore sample before and after immersion in the 3.5 wt% NaCl solution for 14 d. In Figure 8, a thick corrosion product layer generated around the pore obviously, which demonstrates corrosion product is prone to accumulate in pores.

### 3.5. Interpretation of Corrosion

In this work, the corrosion behaviors of the copper samples with different pores in the static and dynamic 3.5 wt% NaCl solutions were evaluated. In the static solution the corrosion resistance of the samples was boosted with the increase in the number of pores (96 > 72 > 24 > 0). By contrast, the 24-pore sample is more susceptible to corrosion than the sample without the pore in the dynamic solution.

Cu → Cu^+^ + e^−^ (1)

Cu^+^ + Cl^−^ → CuCl (2)

CuCl + Cl^−^ → CuCl_2_^−^ (3)

2 CuCl + H_2_O → 2 Cu_2_O + 2 HCl (4)

Cu_2_O + 3 OH^−^ + Cl^−^ → Cu(OH)_3_Cl + CuO (5)

1/2 O_2_ + H_2_O + 2e^−^ → 2 OH^−^ (6)

CuCl + OH^−^ → Cu_2_O + H_2_O + Cl^−^ (7)

In NaCl solution, the anodic polarization process undergoes a copper dissolution reaction. The copper sample dissolves to form Cu^+^, and Cu^+^ combines with Cl^−^ in solution to form CuCl insoluble matter (Equation (2)). The CuCl insoluble matter is unstable and converts to CuCl_2_^−^ soluble substance (Equation (3)), which promotes the further dissolution of the copper. Therefore, the anodic dissolution of the copper sample is mainly controlled by the diffusion of CuCl_2_^−^. When the reaction enters the passivation zone, CuCl can be hydrolyzed to generate Cu_2_O dense oxide film (Equation (4)), which can exist in the film of CuCl to protect the copper matrix. When the copper specimen is in the limiting current region, the Cu_2_O oxide film forming on the sample surface reacts under the action of Cl^−^ to convert to CuO and Cu(OH)_3_Cl (Equation (5)) loose double-layer corrosion structure; in this process, the current of the anode reaction increases with the increase in polarization potential.

As copper sample is soaked in NaCl solution, the cathode undergoes oxygen reduction reaction, and its polarization process is affected by oxygen concentration and oxygen diffusion. The total reaction process occurs, as shown in Equation (6). When the oxygen concentration on the sample surface increases, the reaction formula moves towards a positive direction, the concentration of the generating OH^–^ increases, and unstable CuCl insoluble matter is more likely to form dense Cu_2_O oxide film, thereby increasing the corrosion resistance of the sample.

In the static 3.5 wt% NaCl solution, the corrosion rate of the copper sample is mainly affected by the anodic reaction process. As pores were introduced in the copper sample, the exposed area of the copper matrix in the solution increased, leading to more CuCl on the sample surface under the erosion of Cl^−^. Since the experiment was carried out in an open system, the presence of oxygen in the solution was definite; so Cu_2_O formed on the sample, and CuCl was also hydrolyzed to generate Cu_2_O. Cu_2_O was further converted to Cu(OH)_3_Cl and CuO corrosion products in the NaCl solution. These corrosion products covered the metal surface and tended to accumulate in and around the pores, making it difficult for Cl^−^ in solution to penetrate these oxidation products, thereby reducing Cl^−^ attack to the copper matrix. Therefore, as the number of pores increased, the larger the exposed area, the less corrosion.

In the dynamic 3.5 wt% NaCl solution, on the one hand, the introduction of pore structure raised the specific surface area of the porous sample, leading to the increase in reaction area with Cl^−^ in the solution, so more metal dissolved. At this time, the reaction in Equation (3) moved towards a positive direction. A variety of oxidative corrosion products such as dense Cu_2_O, loose Cu(OH)_3_Cl and CuO were also generated. The flow of the solution caused the corrosion products to fall off easily, thereby reducing the protective function for the copper matrix, so the corrosion of the sample was accelerated. On the other hand, the introduction of the pore structure provided more oxygen diffusion channels, increased the surface oxygen concentration on the copper matrix, and made the cathodic reaction in Equation (6) move towards a positive direction. As a result, OH^−^ concentration in the solution rose, further promoting the conversion of unstable CuCl insoluble matter to dense oxide film Cu_2_O, slowing down corrosion. The combined effect of the two aspects determined the corrosion rate of the porous samples. For the 24-pore specimen the corrosion-accelerated effect by the pores on matrix dissolution was greater than the protective function by boost of oxygen diffusion, so compared with the 0-pore sample, the corrosion current increased and the corrosion aggravated in the 24-pore sample. When the number of pores was further increased, the samples with 72 and 96 pores exhibited almost the same anodic polarization curve as that of the 24-pore sample, indicating that the three kinds of samples possessed basically the same rate of dissolution as reaction with Cl^−^. However, from the cathodic polarization curves, with the increase in the number of pores, oxygen diffusion was easier, and the oxides formed on copper matrix are thicker and denser, which limited the corrosion of the copper matrix.

## 4. Conclusions

In this work, the corrosion behaviors of porous copper in static and dynamic 3.5 wt% NaCl solutions are studied by the corrosion electrochemical and SKP methods, and the following conclusions are obtained:

(1) In the static NaCl solution, the corrosion resistance of copper samples with different pores gradually increased with the rise in the number of pores; when the number of pores was 96, the corrosion resistance was the best.

(2) In the dynamic NaCl solution, the corrosion resistance of the samples decreased first and then increased with the addition of the number of pores; when the number of pores was 24, the corrosion resistance was the worst; the sample with 96 pores still possessed the best corrosion resistance.

(3) The results of the SKP test demonstrated that after immersion in 3.5 wt% NaCl solution for 14 d, the inner surface of the pore has lower possibility to corrode than the outer surface.

## Figures and Tables

**Table 1:** Chemical composition of pure copper T2.

Element	Cu	P	Bi	Sb	Fe	Ni	Pb	Sn	Zn
Content (wt.%)	99.99	0.002	0.001	0.002	0.004	0.0004	0.0002	0.0003	0.0001

**Table 2:** Polarization parameters of pure copper samples with different numbers of pores in the static and dynamic 3.5% NaCl solutions.

Flow Rate of NaCl Solution (m/s)	Samples	OCP (V vs. SCE)	E_corr_ (V vs. SCE)	I_corr_ (μA/cm^2^)	β_a_ (mV)	β_c_ (mV)
0	C-0	−0.242	−0.291	3.445	17.43	14.91
	C-24-I-O	−0.238	−0.287	1.845	21.98	13.09
	C-72-I-O	−0.241	−0.258	1.134	20.25	17.25
	C-96-I-O	−0.239	−0.252	0.967	12.01	12.62
0.17	C-0	−0.252	−0.241	13.88	14.08	5.917
	C-24-I-O	−0.240	−0.213	16.28	21.75	5.563
	C-72-I-O	−0.242	−0.224	11.43	13.56	2.143
	C-96-I-O	−0.245	−0.236	10.37	16.11	4.572

**Table 3:** Electrochemical impedance parameters of samples in the static 3.5 wt% NaCl solution.

Number of Pores	R_s_ (Ω·cm^2^)	Q_f_ Y_01_ × 10^−4^ (Ω^−1^·s^n^ cm^−2^)	n_1_	R_f_ (Ω·cm^2^)	W (Ω^−1^·s^−0.5^cm^−2^)	Q_dl_ Y_02_ × 10^−4^ (Ω^−1^·s^n^ cm^−2^)	n_2_	R_ct_ (Ω·cm^2^)	χ^2^ (10^−3^)
C-0	25.76	0.7963	0.829	85	0.00204	7.103	0.605	1043	0.561
C-24-I-O	19.56	0.5249	0.874	157	0.00165	2.620	0.692	1674	0.709
C-72-I-O	13.79	0.6990	0.853	333	0.00174	2.141	0.657	2293	1.19
C-96-I-O	23.08	0.1594	0.897	492	0.000988	0.8457	0.762	2713	1.47

**Table 4:** Electrochemical impedance parameters of samples in the dynamic 3.5 wt% NaCl solution.

Number of Pores	R_s_ (Ω·cm^2^)	Q_f_ Y_01_ × 10^−4^ (Ω^−1^·s^n^ cm^−2^)	n_1_	R_f_ (Ω·cm^2^)	Q_dl_ Y_02_ × 10^−4^ (Ω^−1^·s^n^ cm^−2^)	n_2_	R_ct_ (Ω·cm^2^)	χ^2^ (10^−3^)
C-0	12.73	18.64	0.665	849	3.492	0.644	972	0.636
C-24-I-O	18.84	36.00	0.747	1345	3.171	0.645	892	1.52
C-72-I-O	18.24	2.782	0.631	1032	14.06	0.643	1756	1.47
C-96-I-O	29.51	61.75	0.943	989	2.958	0.594	1718	2.28

## Data Availability

The data presented in this study are available on request from the corresponding author. The data are not publicly available due to privacy reasons.

## References

[1] Liu P.S., Chen G.F. (2014). Chapter Four—Special Porous Metals. Porous Materials.

[2] Liu P.S., Chen G.F. (2014). Chapter Three—Application of Porous Metals. Porous Materials.

[3] Tang F., Fudouzi H., Uchikoshi T., Sakka Y. (2004). Preparation of porous materials with controlled pore size and porosity. J. Eur. Ceram. Soc..

[4] Nakajima H. (2007). Fabrication, properties and application of porous metals with directional pores. Prog. Mater. Sci..

[5] Singh H., Saxena P., Puri Y.M. (2021). The manufacturing and applications of the porous metal membranes: A critical review. CIRP J. Manuf. Sci. Technol..

[6] Otaru A.J. (2020). Review on Processing and Fluid Transport in Porous Metals with a Focus on Bottleneck Structures. Met. Mater. Int..

[7] Banerjee A., Paul D. (2021). Developments and applications of porous medium combustion: A recent review. Energy.

[8] Tauseef ur R., Ali H.M., Janjua M.M., Sajjad U., Yan W.-M. (2019). A critical review on heat transfer augmentation of phase change materials embedded with porous materials/foams. Int. J. Heat Mass Transf..

[9] Wang Q., Han F., Wu J., Hao G. (2007). Damping behavior of porous CuAlMn shape memory alloy. Mater. Lett..

[10] Guiping C., Deping H., Guangji S. (2001). Underwater sound absorption property of porous aluminum. Colloids Surf. A Physicochem. Eng. Asp..

[11] Long F., Duan Y., Yu S., Jia H., Bu Y., Huang J. (2022). Effect of porous materials on explosion characteristics of low ratio hydrogen/methane mixture in barrier tube. J. Loss Prev. Process Ind..

[12] Goodall R., Chang I., Zhao Y. (2013). 10—Porous metals: Foams and sponges. Advances in Powder Metallurgy.

[13] Sundarram S.S., Li W. (2014). The effect of pore size and porosity on thermal management performance of phase change material infiltrated microcellular metal foams. Appl. Therm. Eng..

[14] Xu Z., Hao H. (2014). Electromagnetic interference shielding effectiveness of aluminum foams with different porosity. J. Alloys Compd..

[15] Guo Y., Liu F., Bian X., Lu K., Huang P., Ye X., Tang C., Li X., Wang H., Tang K. (2022). Effect of Pore Size of Porous-Structured Titanium Implants on Tendon Ingrowth. Appl. Bionics Biomech..

[16] Wan T., Liu Y., Zhou C., Chen X., Li Y. (2021). Fabrication, properties, and applications of open-cell aluminum foams: A review. J. Mater. Sci. Technol..

[17] Li X., Sun M., Rooke J.C., Chen L., Su B.-L. (2013). Synthesis and applications of hierarchically porous catalysts. Chin. J. Catal..

[18] Biswas N., Ding J.L. (2015). Numerical study of the deformation and fracture behavior of porous Ti6Al4V alloy under static and dynamic loading. Int. J. Impact Eng..

[19] Arensburger D.S., Pugin V.S., Fedorchenko I.M. (1968). Corrosion resistance of porous titanium in some aggressive media. Sov. Powder Metall. Met. Ceram..

[20] Seah K.H.W., Thampuran R., Teoh S.H. (1998). The influence of pore morphology on corrosion. Corros. Sci..

[21] Wu L., He Y.-H., Jiang Y., Zeng Y., Xiao Y.-F., Nan B. (2014). Effect of pore structures on corrosion resistance of porous Ni3Al intermetallics. Trans. Nonferrous Met. Soc. China.

[22] Wu S.-H., Li Y., Zhang Y.-Q., Li X.-K., Yuan C.-F., Hao Y.-L., Zhang Z.-Y., Guo Z. (2013). Porous titanium-6 aluminum-4 vanadium cage has better osseointegration and less micromotion than a poly-ether-ether-ketone cage in sheep vertebral fusion. Artif. Organs.

[23] Zhang J., An Y., Ma H. (2022). Research Progress in the Preparation of Aluminum Foam Composite Structures. Metals.

[24] Chen X., Fu Q., Jin Y., Li M., Yang R., Cui X., Gong M. (2017). In vitro studying corrosion behavior of porous titanium coating in dynamic electrolyte. Mater. Sci. Eng. C.

[25] Yao S., Wang D., Cao Y., Li G., Huo Q., Liu Y. (2015). Two stable 3D porous metal–organic frameworks with high performance for gas adsorption and separation. J. Mater. Chem. A.

[26] Bakhsheshi-Rad H.R., Hamzah E., Abdul-Kadir M.R., Daroonparvar M., Medraj M. (2015). Corrosion and mechanical performance of double-layered nano-Al/PCL coating on Mg–Ca–Bi alloy. Vacuum.

